# Physical Activity and Executive Function in Children With ADHD: The Mediating Role of Sleep

**DOI:** 10.3389/fped.2021.775589

**Published:** 2022-01-20

**Authors:** Xiao Liang, Ru Li, Stephen H. S. Wong, Raymond K. W. Sum, Peng Wang, Binrang Yang, Cindy H. P. Sit

**Affiliations:** ^1^Department of Sports Science and Physical Education, The Chinese University of Hong Kong, Hong Kong, China; ^2^The Faculty of Physical Education, Shenzhen University, Shenzhen, China; ^3^Cardiac Rehabilitation Center, Fuwai Hospital, Chinese Academy of Medical Sciences & Peking Union Medical College, Beijing, China; ^4^Children's Healthcare & Mental Health Center, Shenzhen Children's Hospital, Shenzhen, China

**Keywords:** ADHD, physical activity, sleep quality, executive functions (EF), children

## Abstract

This study examined the mediating role of sleep in the relationship between physical activity and executive function in children with attention deficit hyperactivity disorder (ADHD). Fifty-six children with ADHD were recruited from Shenzhen Children's Hospital. Participants wore an accelerometer for seven consecutive days to measure physical activity and sleep quality. Activity counts were analyzed to measure moderate-to-vigorous physical activity (MVPA). Four sleep parameters, including sleep latency (SL), sleep efficiency, total sleep time, and wake after sleep onset were recorded from the actigraph. Three core executive functions, inhibitory control; working memory (WM); and cognitive flexibility (CF), were assessed from computer-based tasks: the flanker task, and the Tower of London and Trail Making Tests, respectively. The regression results showed that MVPA was negatively associated with SL (−0.169; 95%CI [−0.244, −0.112]). WM (total scores) was positively related to MVPA (0.028, 95%CI [0.008, 0.048]), but negatively related to SL (−0.105, 95%CI [−0.167, −0.030]). CF (part B errors) was negatively associated with MVPA (−0.031, 95%CI [−0.055, −0.005]) and positively correlated with SL (0.184, 95%CI [0.092, −0.260]). The indirect effect of SL was found for MVPA and WM (0.018, 95%CI [0.015, 0.034]), supporting the indirect partial mediation. Similarly, the indirect effect of SL was found between MVPA and CF (−0.031, 95%CI [−0.060, −0.012]), supporting the indirect partial mediation. The mediating role of SL in children with ADHD suggests that the intensity of physical activity plays a key role in linking sleep quality and executive function in this group.

## Introduction

Attention deficit hyperactivity disorder (ADHD) is a neurodevelopmental condition, commonly diagnosed in childhood (1), with a global prevalence of 7.2% among children and adolescents (2). ADHD is generally characterized by age-inappropriate behaviors, including inattention, hyperactivity, and/or impulsivity (3). Disorder-related symptoms are associated with different levels of problems, including physical inactivity (4), executive dysfunction (5) and sleep problems (6).

Executive functions (EFs) are a set of cognitive skills that are involved in top-down control processes used in planning, organizing, and monitoring complex, goal-directed behaviors (7). EFs distinguish between three core functions [inhibitory control (IC), working memory (WM), and cognitive flexibility (CF)] and higher-level functions (e.g., reasoning, planning, and problem-solving) (8). EF skills are indispensable for all ages, including the school performance of children and adolescents (8), sleep duration and quality (9, 10), and physical and mental health (11). Moreover, EF is a higher-order cognitive function (12) that contributes to successful learning in school, management of stress-related activities, and inhibition of inappropriate behaviors in the daily lives of children with ADHD (13).

Physical activity (PA) has emerged as a promising compensation method that can positively affect cognitive function from early childhood (14) to adulthood (15) and can be used to reduce the risk of age-related cognitive decline (16). Studies have documented that for children and adolescents, enhanced cognitive functioning resulting from PA was most clearly seen in EFs (7, 17). Furthermore, preliminary evidence suggests that participation in regular PA is associated with reduced severity of ADHD symptoms (18), the development of motor proficiency (19) and improved sleep problems (20). These documented beneficial effects may be due to improved EFs that are in turn due to PA engagement (7, 21). However, there is still limited understanding of the mechanisms by which PA exerts its effects on human cognition (22), especially with children with neurodevelopmental disorders (e.g., ADHD and autism spectrum disorder).

Among the numerous potential mechanisms for the relationship between PA and cognition, sleep has been proposed as one possible mechanism (23). Participation in regular PA and exercise may facilitate sleep regulation, and sleep can lead to enhanced cognitive functioning. Sleep is an essential health indicator for children (24). Another study found a positive relationship between sleep patterns and PA levels (25). These findings, however, are generally observed in individuals who do not have disabilities and are not at risk of sleep problems. Seventy percent of children with ADHD experience sleep problems frequently, including bedtime resistance, night waking, and daytime sleepiness (26–28). Indeed, sleep problems may exacerbate existing ADHD symptoms, academic impairment, and adverse effects on health-related quality of life for children with ADHD and their families (29, 30). Poor sleep (e.g., short total sleep time) has been associated with poorer WM in young adults without disabilities (10) and parent-reported sleep problems (bedtime resistance) have been associated with poorer WM in children with ADHD (31).

**Figure 1:**
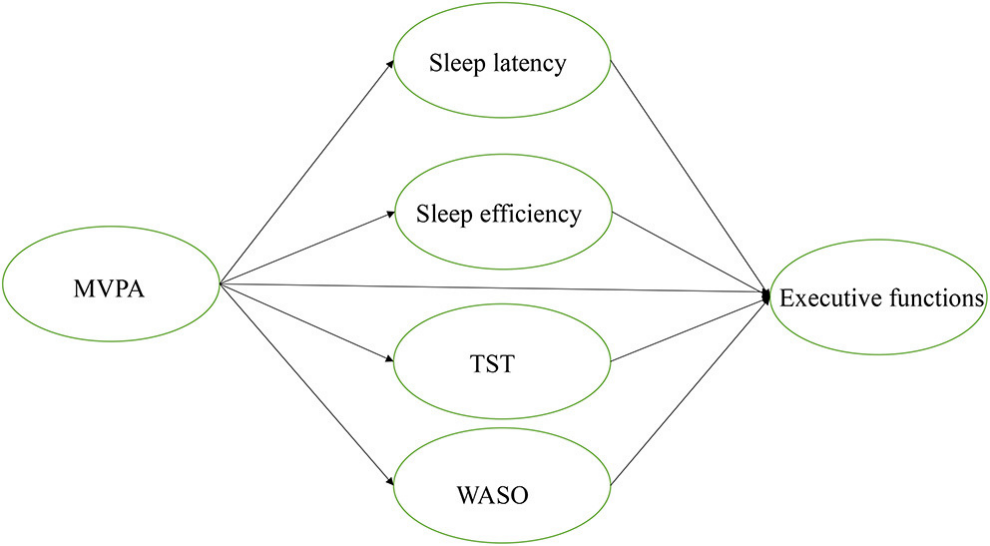
Hypothesized model. MVPA, moderate to vigorous physical activity, TST, total sleep time; WASO, waking after sleep onset.

PA and sleep are positively related to cognition, especially in EFs (17). One recent review found that chronic exercise interventions with moderate levels of PA promoted EFs in children and adolescents with ADHD (32). However, it is unclear if there are mechanisms (e.g., mediators) linking PA and EFs among ADHD populations, especially children. Scant attention has been paid to the role of sleep in understanding cognitive performance (e.g., IC, WM, and CF) and the relationships among PA, sleep, and EFs in children with ADHD. Given the known positive relationships among PA, sleep, and WM (10, 25), this study expected that (i) PA would be positively associated with sleep quality; (ii) sleep quality would, in turn, be positively linked with EFs; and (iii) sleep quality would mediate the relationship between PA and domains of EFs. The proposed model is illustrated in Figure 1.

## Materials and Methods

### Participants

Children aged 6–12 years old and diagnosed with ADHD were recruited from local children's hospital. The inclusion criteria was a diagnosis of ADHD by psychiatrists based on the *Diagnostic and Statistical Manual of Mental Disorders, Fifth Edition* (3). They had to have a neuropsychiatric interview with their parents by a psychiatrist based on the *Schedule for Affective Disorders and Schizophrenia for School-Age Children-Present and lifetime Version* (33, 34). Exclusion criteria were; other comorbid psychiatric or neurological disorders and a full-scale IQ of less than 80, as measured by the *Chinese Wechsler Intelligence Scale for Children, fourth edition* (35). Finally, 56 children (M_age_ = 8.82, SD = 1.49, 84% boys) with ADHD who met all inclusion criteria were included. Written consent was obtained from the children's parents or guardians. This study design complies with the Declaration of Helsinki ethical standards and was approved by the Survey and Behavioral Research Ethics Committee, The Chinese University of Hong Kong (Reference No. SBRE-19-244).

### Study Design and Procedures

This study utilized a cross-sectional design. Participants were instructed to complete three computer-based EF tasks and self-reported questionnaires. Instructions for wearing an actigraphy accelerometer and for filling in a sleep log, assisted by their parents, were provided to participants. Each participant completed all computer tasks individually assisted by trained research assistants; there were practice trials for each task to ensure that the instructions were understood by the participants. To determine body mass index (BMI), participants' height and weight were measured after the tasks.

### PA Measures

#### Objectively Measured PA (Actigraph GTX3)

Children's PA levels were assessed using accelerometry (GT3X model; ActiGraph, Pensacola, FL), an objective and widely used PA measure with children with ADHD (36–38). All participants were asked to wear the accelerometer around the non-dominant wrist for seven consecutive days (5 weekdays and 2 weekend days), follow their daily routines and remove the accelerometer only for bathing or swimming. Activity counts were analyzed to determine step counts per minute, the proportion of time spent in moderate-to-vigorous PA (MVPA).

### Cognitive Tasks

EFs were assessed through the following cognitive tasks. All three computer tasks were validated for assessing EFs in children with ADHD summarized by previous review (32). These instruments were administered *via Inquisit Lab*^*TM*^ *5* (Millisecond Software, Inc., Seattle, WA, USA).

#### Flanker Task

IC was measured using a computer-based Eriksen flanker task (39). In the task, target stimuli are arrows pointing to the right or the left, presented at the center of the screen. The target arrow's direction indicates whether the child needs to press the left or the right response button. Two distracters surround the target stimulus on both sides (left and right). Two trial types were used: congruent and incongruent. Participants were asked to respond to a central arrow's direction in either congruent or incongruent trials as quickly and as accurately as possible. In a congruent trial, the target arrow was flanked by arrows pointing in the same direction as the target, whereas in incongruent trials, the flankers pointed in the opposite direction. Each participant was engaged in the experiment for ~5 mins. The outcome measures of task performance were reaction-time and response-accuracy in congruent and incongruent conditions. Short reaction-time and high response accuracy indicated better IC.

#### The Tower of London

The Tower of London test was used to assess WM and problem-solving skills (40) through the movement of three different colored balls on three different-sized pegs. Each participant was required to rearrange the three colored balls (red, blue, and green) to match a target picture shown on the screen. Before the practice trial, each participant was informed that only one ball could be moved at a time and that the underneath ball could not be moved if another ball was on top of it. The goal of the test is that the colored balls should be positioned using a minimum number of moves to achieve the target position. Participants were allowed to reset a round of the game if they realized making a mistake. This task consisted of 12 trials and progressed from easier trials (could be completed with a minimum of two moves) to more difficult trials (could be completed with a minimum of five moves). Total score, defined as the sum of the individual problem scores, was recorded; the maximum achievable score was 36.

#### Trail Making Test

CF was measured using a computer-based Trail Making Test (41). This test was performed in two parts: part A (numbers only) and part B (letters and numbers), with each part including a trial. Before each trial, each participant was instructed to complete part A by connecting numbers in order (e.g., 1, 2, 3) to 25, and to finish part B by connecting each number to the corresponding letter in numerical and alphabetical order (e.g., 1-A-2-B-3-C, etc.) to 13, using the mouse. The Trail Making Test performance was recorded by errors and the total completion time in part A and part B, respectively. A shorter total completion time and fewer errors indicated a better performance.

### Sleep Measures

#### Objectively Measured Sleep Quality

Four sleep parameters, including sleep latency (SL, length of time in minutes to fall asleep), sleep efficiency (SE, the percentage of actual sleep time divided by the time between sleep onset and sleep offset), total sleep time (TST, actual sleep time), and wake after sleep onset (WASO, length of wake time in minutes between sleep onset and sleep offset) were measured using an actigraph accelerometer and a sleep-log book. Participants' parents were asked to record the sleep onset, sleep offset, and total sleep length in a sleep log during assessment week. The accelerometer has been widely used to measure sleep in children with and without ADHD (38). The Sadeh algorithm (42), the most commonly used algorithm for sleep–wake scoring in children, was implemented to identify sleep onset and sleep offset (43).

### Statistical Analysis

Descriptive statistics were calculated to characterize the sample, regarding information on participants' gender, age, BMI, and ADHD symptoms. Skewness and Kurtosis tests were performed to assess if the data were normally distributed. Bivariate correlations among the variables were calculated Using Mplus (version 7.0), a path analysis with a maximum likelihood estimation was conducted to explore the overall direct and indirect relationships among the variables. A bootstrapping method was used for estimating direct and indirect effects with multiple mediators (SL, SE, TST, and WASO) in which the effects of PA on EFs were mediated through four mediators. All statistical tests were two-tailed, and significance was set at *p* < 0.05. A 5,000-bootstrapping method was used to test the significance of the total and indirect effects. The 95% confidence intervals for the coefficients calculated by bootstrapping methods were considered statistically significant if the confidence intervals did not include zero.

## Results

### Participant Characteristics

Of the 59 participants, 3 were excluded from analysis due to: (1) missing accelerometer data (*n* = 1) or (2) having fewer than five valid days (four weekdays and one weekend day) of accelerometer data (*n* = 2). Of the remaining 56 participants (9 girls; 47 boys), 25 were diagnosed with ADHD-I (inattention), 7 with ADHD-H (hyperactivity), and 24 with ADHD-C (combined)(Table 1).

**Table 1:** Demographic and physical characteristics of the participants (*n* = 56).

**Characteristics**	**Mean(±SD)/N (%)**
Gender	47 boys (83.9%), 9 girls (16.1%)
Age (years)	8.82 (± 1.49)
Weight (kg)	30.79 (± 10.13)
Height (cm)	132.86 (± 11.40)
BMI (kg/m^2^)	17.01 (± 3.15)
Type of ADHD	ADHD-H 7 (12.5%) ADHD-I 25 (44.6%) ADHD-C 24 (42.9%)

*BMI, body mass index; ADHD-H, attention-deficit hyperactivity disorder-hyperactivity; ADHD-I, attention-deficit hyperactivity disorder-inattention; ADHD-C, attention-deficit hyperactivity disorder-combined*.

### Preliminary Analysis

As shown in Table 2, MVPA was negatively correlated with the sleep latency, positively correlated with working memory (total scores), and negatively related with errors of cognitive flexibility. Regrading the sleep quality, the sleep latency was negatively related to the working memory (total scores), and positively related to errors of cognitive flexibility. All significant levels reached at *p* < 0.05.

**Table 2:** Correlations among MVPA, sleep quality, and executive functions.

	**Mean**	**SD**	**1**	**2**	**3**	**4**	**5**	**6**	**7**	**8**	**9**	**10**	**11**	**12**	**13**	**14**
1. MVPA (mins)	87.34	38.25	–													
2. SL (mins)	20.29	12.34	−0.53^**^	–												
3. SE (%)	78.62	0.06	−0.40	−0.143	–											
4. TST (mins)	408.03	30.66	−0.13	0.07	0.75^**^	–										
5. WASO (mins)	92.98	38.00	0.20	−0.17	−0.94^**^	−0.68^**^	–									
6. C-ACC (%)	0.975	0.058	−0.01	−0.03	−0.09	−0.03	0.10	–								
7. IC-ACC (%)	0.933	0.148	−0.10	0.12	−0.21	−0.07	0.18	0.66^**^	–							
8. C-RT (sec)	853.98	370.27	−0.04	−0.09	0.02	0.05	0.02	−0.09	−0.30^*^	–						
9. IC-RT (Sec)	1,046.29	502.35	−0.03	−0.05	−0.003	−0.05	0.007	−0.28^*^	−0.39^*^	0.86^*^	–					
10. WM (total)	27.82	3.40	0.48^**^	−0.53^**^	−0.05	−0.23	0.19	−0.04	−0.08	−0.02	0.03	–				
11. CF-error A	2.16	2.14	0.12	0.07	−0.03	−0.03	0.005	−0.19	−0.18	0.31^*^	0.35^**^	−0.20	–			
12. CF-CTA (sec)	134,526.35	56,382.68	0.06	0.12	−0.17	−0.11	0.13	−0.13	−0.22	0.52^**^	0.57^**^	−0.03	0.59^**^	–		
13. CF-error B	5.22	4.58	−0.52^**^	0.63^**^	0.05	0.20	−0.22	−0.20	−0.21	0.15	0.20	−0.50^**^	0.09	0.27^*^	–	
14. CF-CTB (sec)	222,304.38	11,2997.68	−0.03	0.11	−0.13	0.003	0.12	−0.10	−0.22	0.54^**^	0.54^**^	−0.14	0.35^**^	0.70^**^	0.47^**^	–

* *p < 0.05 and*

** *p < 0.01*.

*MVPA, moderate-to-vigorous physical activity; SL, sleep latency; SE, sleep efficiency; TST, total sleep time; C-ACC, accuracy rate in the congruent condition; IC-ACC, accuracy rate in the incongruent condition; C-RT, reaction time in the congruent condition; IC-RT, reaction time in the incongruent condition; WM, total scores of working memory; CF-error A, errors of part A of cognitive flexibility; CF-CTA, completion time of part A of cognitive flexibility; CF-error B, errors of part B in the cognitive flexibility; CF-CTB, completion time of part B of cognitive flexibility*.

### Path Analysis

The hypothesized model (Figure 1) was tested to examine the direct relationships among MVPA, sleep quality (SL, SE, TST, and WASO), and Efs (IC, WM, and CF). The mediating role of sleep quality in MVPA and EF was also investigated.

**Figure 2:**
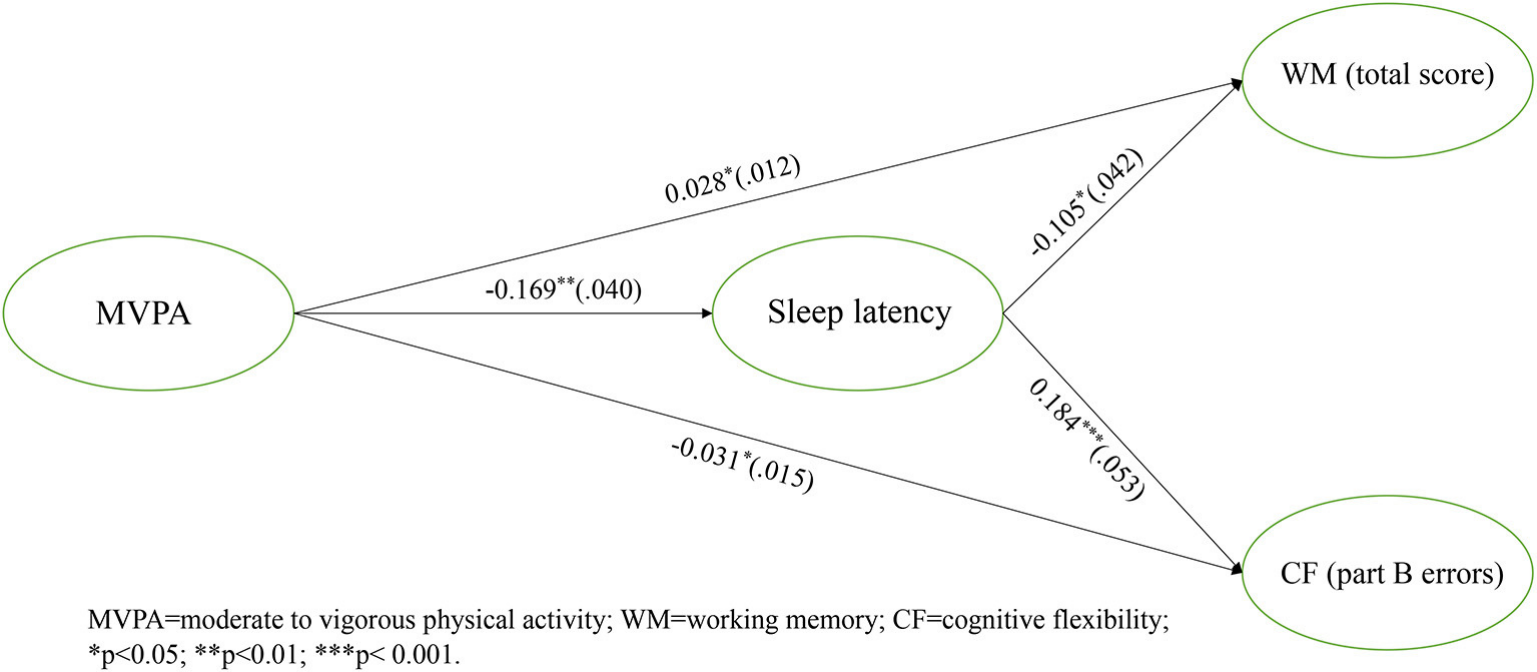
Final model with significant standardized estimates and standard errors. MVPA, moderate to vigorous physical activity; WM, working memory; CF, cognitive flexibility; *p < 0.05; **p < 0.01; ***p < 0.001.

The regression results showed that MVPA was associated with WM and CF *via* the mediation of SL. Specifically, MVPA was negatively associated with SL (−0.169; 95%CI [−0.244, −0.112]). WM (total scores) was positively related to MVPA (0.028, 95%CI [0.008, 0.048]) but negatively related to SL (−0.105, 95%CI [−0.167, −0.030]). CF (part B errors) was negatively associated with MVPA (−0.031, 95%CI [−0.055, −0.005]) and positively correlated with SL (0.184, 95%CI [0.092, −0.260]). The regression results for testing mediation were showed in Figure 2. These results support that sleep latency partial mediated the association between MVPA and WM (indirect effect = 0.018, *SE* = 0.009, 95%CI [0.002, 0.038]). Similarly, the indirect effect of SL was revealed in the link between MVPA and CF (part B errors) (indirect effect = −0.031, *SE* = 0.014, 95%CI [−0.065, −0.008]), supporting the indirect partial mediation. The mediating effect of sleep quality between MVPA and IC was not supported (Figure 2).

## Discussion

We examined the relationships between MVPA and EFs to better understand the mediating role of sleep quality on MVPA and EFs among children with ADHD. We observed that with greater amounts of MVPA during the daytime, participants' SL decreased. This is a novel finding, as previous studies have shown that increasing PA may promote sleep quality (44) and that high PA levels would be expected to show healthy sleep patterns (45). However, these findings were based on typically-developing children without sleep problems. Using the objective measurement of sleep quality and PA, our results extend the understanding about previous analysis of healthy children and focus more on PA intensity in children with ADHD. The latest *WHO Guidelines on Physical Activity and Sedentary Behaviors* recommend that children and adolescents with disabilities should engage in at least 60 mins of MVPA daily, primarily aerobic PA, throughout the week (46). A previous study also indicated that higher daytime activity is associated with shorter SL. More specifically, vigorous PA was significantly related to a decrease in SL (47). Prior review studies also found that the level of daytime exercise was a critical factor influencing the length of SL in children (48). Higher amounts of PA and aerobic exercise have been associated with increased sleep quality ratings (23). Therefore, PA intensity should be considered in designing exercise intervention studies to improve sleep quality in children with ADHD.

Furthermore, SL significantly predicted EF performance in the participants. A longer duration of SL was significantly associated with poorer performance in WM and CF. In contrast, we observed no predictive association between SL and inhibition. Thus, sleep quality might be a critical contributor to the development of EFs, as reported in previous studies showing that high sleep quality leads to better cognitive performance (22, 49). Another study reported a relationship between longer sleep-onset latency and poorer WM in school-aged children with typical development (50). Similarly, studies focusing on children with ADHD reported that bedtime resistance and sleep-onset delay were associated with poorer WM (31, 51).

Our results also strengthen the association between SL and WM in children with ADHD. Another novel finding was that longer SL duration was significantly related to poorer CF in children with ADHD. Previous studies investigating CF are rare in both children with ADHD and their typically-developing peers (52). This is due to the fact that CF is frequently associated with WM and inhibition in early childhood, but gradually improves until adolescence (53). Furthermore, few studies have investigated the relationship between sleep quality and CF in children with and without ADHD. Only one recent study reported an association between poorer sleep quality and poorer CF performance, as measured by the Stroop test in healthy older adults (54). Cognitive inflexibility was frequently observed in individuals with ADHD; (52, 55) this specific EF domain is related to learning and academic readiness (56), as well as predicted social understanding from middle childhood (57). Therefore, CF is essential for children with ADHD, and appropriate interventions targeting sleep quality and CF should be provided.

This study is the first to report that SL significantly mediates cross-sectional associations between MVPA and WM and CF. These novel findings are consistent with the broad view that poor sleep can have serious consequences on cognition (58) and that this process may be improved by PA (17). To date, only one study has included objectively measured PA, sleep, and cognition in one model to test if sleep quality (SE/TST) can account for the relationship between PA and executive control in young and older adults (17). The results of that study reported that SE, rather than TST, significantly mediated the relationship between PA and WM, switching, verbal fluency, and processing speed (17).

Our findings are consistent with the view that sleep quality changes are linked to both PA and cognition (22). The mechanisms underlying the relationships among PA/exercise, sleep, and cognition are unclear. One possible explanation is that PA stimulates changes in body temperature and produces melatonin before sleep, resulting in later changes in body thermoregulation during sleep cycles (59). The increasing body temperature during exercise promotes higher melatonin production, which decreases sleep onset and improves sleep quality and quantity further (23). Another possible explanation for mechanisms among PA, sleep quality and cognitive functions may be the cerebral blood flow. PA may boost the cerebral blood flow regulation, resulting in improvement of cognitive processing by a change in cerebral oxygenation during sleep cycles throughout the day (60, 61). One recent study focusing on children with autism reported that acute exercise with MVPA levels of intensity showed a change in cerebral oxygenation and IC (62). This change in cerebral blood pressure may better explain an association between PA and EFs by improved sleep quality (23). Therefore, examining this relationship among PA, sleep, and cognition is critical and has clinical implications. Determining the optimal intervention for exercise may improve sleep health and cognitive development, as well as lead to the discovery of a method to reverse executive dysfunctions in ADHD populations.

The current study benefited from several strengths (e.g., the use of an actigraph to objectively measure PA and sleep and the use of computer tasks to measure Efs). However, several limitations should be acknowledged. First, the sample size was relatively small to detect mediation. The recommended sample size to test a small to moderate mediation effect using the bootstrapping method should include 50–100 participants (22). Second, as this study adopted a cross-sectional design, causal mediation could not be established. Future exercise-intervention studies should be conducted to test the hypotheses used in this study. Third, the sleep-onset and sleep-offset times were determined by the sleep diary recorded by participants' parents. Although the actigraph was valid in measuring PA and sleep, it is possible that the results may have been influenced by an underestimation or overestimation of sleep or PA. Lastly, considering sleep latency, as the only significant mediator in this study, was probably the most vulnerable indicator affected by core ADHD symptoms (inattention and hyperactivity) compared to other sleep metrics (e.g., sleep efficiency, total sleep time). The potential confounding role of ADHD symptom severity might mediate the effects of MVPA on EF through sleep latency. However, considering the unbalanced distribution of ADHD subtypes underlying this disorder, it is difficult to conduct subgroup analysis to detect whether the effects are robust for specific subgroups.

## Conclusion

SL was found to mediate statistically the significant relationships between MVPA and two core aspects of EF (WM and CF). This mediating role of SL was observed in children with ADHD, suggesting that the intensity of PA plays a key role in linking sleep quality and EF in children with ADHD. Exercise interventions with higher levels of intensities (e.g., MVPA) should be provided for children with ADHD to improve sleep quality and EFs.

## Data Availability Statement

The original contributions presented in the study are included in the article/supplementary material, further inquiries can be directed to the corresponding author/s.

## Ethics Statement

The studies involving human participants were reviewed and approved by the Survey and Behavioral Research Ethics Committee, the Chinese University of Hong Kong (Reference No. SBRE-19-244). This study design complies with the Declaration of Helsinki ethical standards. Written informed consent to participate in this study was provided by the participants' legal guardian/next of kin.

## Author Contributions

XL and CS were responsible for the conceptualization, investigation, and hypothesis of the research. XL and RL conducted the data collection and completed all statistical analyses. CS, SW, RS, PW, and BY reviewed and edited initial draft and its revisions. All authors read and approved the final manuscript.

## Funding

This study was partially funded by Sanming Project of Medicine in Shenzhen the ADHD research group from Peking University Sixth hospital, SZSM201612036.

## Conflict of Interest

The authors declare that the research was conducted in the absence of any commercial or financial relationships that could be construed as a potential conflict of interest.

## Publisher's Note

All claims expressed in this article are solely those of the authors and do not necessarily represent those of their affiliated organizations, or those of the publisher, the editors and the reviewers. Any product that may be evaluated in this article, or claim that may be made by its manufacturer, is not guaranteed or endorsed by the publisher.
